# Genetic variations in *TAS*2*R3* and *TAS*2*R4* bitterness receptors modify papillary carcinoma risk and thyroid function in Korean females

**DOI:** 10.1038/s41598-018-33338-6

**Published:** 2018-10-09

**Authors:** Jeong-Hwa Choi, Jeonghee Lee, Sarah Yang, Eun Kyung Lee, Yul Hwangbo, Jeongseon Kim

**Affiliations:** 1 Department of Cancer Biomedical Science, Graduate School of Cancer Science and Policy, National Cancer Center, 323 Ilsan-ro, Ilsandong-gu, Goyang-si, Gyeonggi-do 10408 Korea; 2 Department of Food Science and Nutrition, Keimyung University, 1095, Dalgubeol-daero, Dalseo-gu, Daegu 42601 Korea; 3 Complex Disease & Genome Epidemiology Branch, Department of Epidemiology, School of Public Health, Seoul National University, 1 Gwanak-ro, Gwanak-gu, Seoul 08826 Korea; 4 Center for Thyroid Cancer, National Cancer Center Hospital, National Cancer Center, 323 Ilsan-ro, Ilsandong-gu, Goyang-si, Gyeonggi-do 10408 Korea

## Abstract

Type 2 taste receptors (T2Rs, *TAS2Rs*) mediate bitterness perception and are involved in diverse defence mechanisms in extraoral tissues. The thyrocyte-expressed T2Rs control thyroid hormone production, and this regulatory role may be associated with susceptibility to thyroid diseases. This study examined whether the variations in *TAS2Rs* modify the risk of papillary thyroid carcinoma (PTC) and whether such T2R-related PTC risk is associated with genetically modified thyroid function. We conducted a case-control study with 763 Korean females, including 250 PTC cases. Seventy-three single-nucleotide polymorphisms in 13 *TAS2R* genes and the pre-diagnosis levels of 4 thyroid-related functional markers [total triiodothyronine (TT3), free thyroxine, thyroid-stimulating hormone and thyroglobulin] were analysed. Individuals with *TAS2R3/4* CC haplotype (rs2270009 and rs2234001) were at a lower risk for PTC than those with the remaining haplotypes (odds ratio = 0.59, 95% confidence interval: 0.36–0.97). Furthermore, TT3 levels were significantly reduced for *TAS2R3/4* CC haplotype carriers compared with other haplotype carriers (p = 0.005). No other genetic variants exhibited critical associations with the PTC phenotype and biomarkers. In summary, genetic variations in T2R3/4 bitterness receptors may modify the PTC risk, and the genetically modified thyroid hormone level by those variations may be linked with the PTC-T2Rs association.

## Introduction

Thyroid centres in the endocrine system regulate human development, homeostasis and metabolism. Therefore, functional perturbation of the thyroid may be associated with subsequent abnormalities in various tissues, including thyroid diseases^1–3^.

Thyroid cancer is one of the most prevalent malignancies in Koreans^4^. The incidence rate of thyroid cancer has been rapidly increasing by approximately 25% over the last decade, making it one of the highest rates worldwide^5^. Although over-diagnosis resulting from advanced detection techniques cannot be dismissed^5^, some factors are reported to be associated with such thyroid health issues. Alterations in environmental factors, including iodine and calcium intake and radiation exposure^6–8^, genetic factors, such as single-nucleotide polymorphisms (SNPs) in the regions of *FOXE1, NRG1* and *NKX2-*1, and their combined effects are all thought to contribute to a high risk of thyroid cancer^9^.

Type 2 taste receptors (T2Rs, *TAS2Rs*) are a class of G protein-coupled receptors (GPCRs) involved in signal transduction on the cellular membrane, especially in response to bitter-tasting compounds. In humans, *TAS2R* genes are located on chromosomes 5, 7 and 12 and encode approximately 25 different T2R isoforms. These receptors are expressed in oral tissues, where the bitterness sensing of food- or water-borne compounds begins, and in other extraoral tissues, including those in the respiratory, genitourinary, gastrointestinal and nervous systems^10^. Universally expressed T2Rs differentiate beneficial or noxious exogenous and endogenous molecules and activate subsequent processes to utilize or eliminate those stimuli^11^, therefore possibly regulating subsequent metabolism and disease risk^12^.

Growing evidence suggests that T2Rs play a role in disease aetiology, with genetic variants serving as modifying factors. The association between *TAS2R38* diplotype and gastrointestinal cancer has been reported in multiple ethnic groups; however, the precise underlying mechanisms are not fully understood to date^13–15^. A pathogenic role of the genetic variations in bitterness receptors was also evident in the exterior of alimentary systems. Altered bitterness receptor functions are associated with susceptibility to cavities, asthma, respiratory infection and nephropathy^16–18^. Studies have also expanded to other isoforms of T2Rs beyond T2R38. The differential expression of T2R4 was observed between cancerous and non-cancerous breast cell line models^19^. The biochemical and pharmacological consequences resulting from coding variations of *TAS2Rs* have been major research interests^20^. Additionally, many agonists responding to T2Rs, including T2R8, T2R10 and T2R14, have been revealed and have shown anticancer effects or associations with anticancer stemness and anti-invasive activity^21–23^.

Recently, the expression of T2Rs and their mechanisms of action were identified in the thyroid^24^. Thyrocyte-expressed T2Rs, especially T2R4, T2R10 and T2R43, regulated the production of hormones via thyroid-stimulating hormone (TSH)-dependent intracellular calcium and iodine efflux. One common variant allele of *TAS2R42* has also been associated with serum concentrations of free triiodothyronine (T3) and thyroxine (FT4)^24^. Given these findings, it is possible to conjecture that genetic variations in *TAS2Rs* modify the risk for thyroid malignancy. Furthermore, the alterations in thyroid function due to such *TAS2R* genetic variants may be associated with its underlying mechanism of action. However, additional epidemiological evidence is required to verify this hypothesis.

In this study, we examined whether *TAS2R* genetic variations may modify the susceptibility to thyroid cancer in Koreans. Additionally, we evaluated whether genetically modified thyroid function by *TAS2R* variations may be associated with such modified risks for thyroid cancer. As biomarkers indicative of thyroid-related function, the following four types of indices were selected and examined: total T3 (TT3); FT4; TSH; and thyroglobulin (Tg). Gender disparities have been observed in thyroid cancer^25^. Furthermore, each subtype of thyroid cancer shows numerous biologically distinctive features^26,27^. Therefore, for a more unbiased approach and better interpretation of the findings, the study was conducted in pathologically homogeneous subjects, including female controls and females with papillary thyroid carcinoma (PTC), the most common type of thyroid cancer in Korea. In addition, biomarkers were evaluated using samples obtained prior to diagnosis.

## Results

### General characteristics of the study subjects

Table 1 presents the descriptive data of subjects analysed in the current study. The cases and controls did not exhibit differences in their analysed anthropometric, life style, socio-economical or reproductive characteristics, with the exception of family history of thyroid cancer. PTC cases were more likely to have a family member with thyroid cancer than controls. Therefore, family history of thyroid cancer was considered a covariate in the subsequent analyses.

**Table 1:** General characteristics of the study subjects.

	All subjects (*n* = 763)	Controls (*n* = 513)	Cases (*n* = 250)	P^a^
Age (years, mean ± SD)	48.9 ± 8.48	48.9 ± 8.51	48.9 ± 8.43	0.955
Body mass index (kg/m^2^)				0.346
<23	402 (52.7)^b^	278 (54.2)	124 (49.6)	
23 to <25	177 (23.2)	117 (22.8)	60 (24.0)	
≥25	179 (23.5)	113 (22.0)	66 (26.4)	
Missing	5 (0.66)	5 (0.97)	.	
Education level				0.646
Elementary school or less	60 (7.86)	39 (7.60)	21 (8.40)	
Middle school	61 (7.99)	39 (7.60)	22 (8.80)	
High school	319 (41.8)	216 (42.1)	103 (41.2)	
College or more	267 (34.9)	189 (36.8)	78 (31.2)	
Missing	56 (7.34)	30 (5.85)	26 (10.4)	
Monthly household income (10,000 won)				0.255
<200	120 (15.7)	81 (15.7)	39 (15.6)	
200 to <400	208 (27.3)	135 (26.3)	73 (29.2)	
400 to <700	217 (28.4)	160 (31.2)	57 (22.8)	
≥700	81 (10.6)	55 (10.7)	26 (10.4)	
Missing	137 (17.9)	82 (15.9)	55 (22.0)	
Marital status				0.445
Married	644 (84.4)	439 (85.6)	205 (82.0)	
Unmarried	21 (2.75)	15 (2.92)	6 (2.40)	
Divorced/Widowed	72 (9.44)	44 (8.58)	28 (11.2)	
Missing	26 (3.41)	15 (2.92)	11 (4.40)	
Alcohol drinking				0.096
Never drinkers	420 (55.1)	271 (52.8)	149 (59.6)	
Ever drinkers	317 (41.6)	223 (43.5)	94 (37.6)	
Missing	26 (3.41)	19 (3.70)	7 (2.80)	
Smoking				0.789
Never smokers	675 (88.5)	449 (87.5)	226 (90.4)	
Ever smokers	69 (9.04)	47 (9.16)	22 (8.80)	
Missing	19 (2.49)	17 (3.31)	2 (0.80)	
Family history of thyroid cancer (first-degree relative)				0.005
No	719 (94.2)	491 (95.7)	228 (91.2)	
Yes	28 (3.67)	12 (2.34)	16 (6.40)	
Missing	16 (2.10)	10 (1.95)	6 (2.40)	
Age at menarche (years)				0.849
≤13	170 (22.3)	114 (22.2)	56 (22.4)	
14–15	294 (38.5)	202 (39.4)	92 (36.8)	
≥16	281 (36.8)	187 (36.5)	94 (37.6)	
Missing	18 (2.36)	10 (1.95)	8 (3.20)	
Postmenopausal: Yes	358 (46.9)	234 (45.6)	124 (49.6)	0.229
Age at menopause (years)				0.608
<46	80 (22.4)	53 (22.7)	27 (21.8)	
46 to <50	122 (34.1)	83 (35.5)	39 (31.5)	
50 to <52	70 (19.6)	41 (17.5)	29 (23.4)	
≥52	70 (19.6)	46 (19.7)	24 (19.4)	
Missing	16 (4.47)	11 (4.70)	5 (4.03)	
Postmenopausal hormone use (ever)				0.995
Yes	116 (32.4)	76 (32.5)	40 (32.3)	
No	209 (58.4)	137 (58.6)	72 (58.1)	
Missing	33 (9.22)	21 (8.97)	12 (9.68)	
Parity				0.534
Yes	676 (88.6)	455 (88.7)	221 (88.4)	
No	46 (6.03)	33 (6.43)	13 (5.20)	
Missing	41 (5.37)	25 (4.87)	16 (6.40)	

^a^p-values for age from Student’s t-test, otherwise from chi-squared tests. ^b^Numbers in brackets represent percentages. SD, standard deviation.

### Genotype/diplotype distribution and the association with PTC

**Figure 1:**
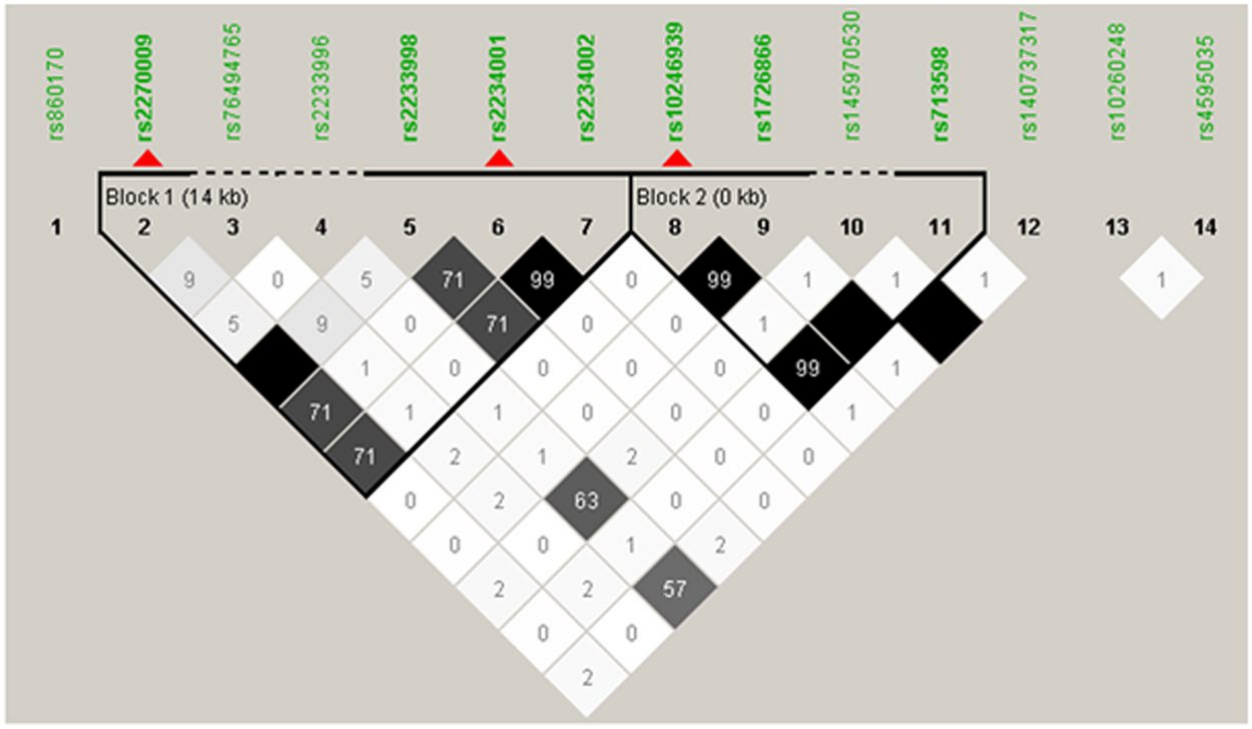
Linkage disequilibrium patterns and tagging single-nucleotide polymorphisms of *TAS2Rs* in chromosome 7. Red triangles denote the tagging loci. The numbers in the squares are *r*^*2*^ values (×100) between the loci, with darker shades indicating greater *r*^*2*^ values.

A total of 73 SNPs in 13 *TAS2R* genes on chromosomes 7 and 12 were obtained as a result of genotyping and imputation (see Supplementary Table S1 for the full list of SNPs). Haplotype-based analysis could provide more comprehensive information for the individual’s genetic background than multiple individual-locus tests. Therefore, we assessed linkage disequilibrium (LD) patterns and tagging loci among the genetic variations for the subsequent analyses. According to Haploview^28^, two LD blocks were evident across 14 SNPs in 6 *TAS2Rs* on chromosome 7 (Fig. 1). Two tagging SNPs in *TAS2R3* (rs2270009) and *TAS2R4* (rs2234001) in the first LD block and 1 tagging locus in *TAS2R38* (rs10246939) in the second LD block were identified. In chromosome 12, a single LD block existed, with 2 taggers (rs10772397 and rs1868769) in 2 *TAS2R* genes (*TAS2R50* and *TAS2R48*) (Supplementary Fig. S1). These tagging SNPs in each LD block were applied to compute the diplotype for the subsequent analyses, with the exception of the *TAS2R38* variants. However, the *TAS2R38* diplotype consists of three loci (rs10246939, rs1726866 and rs713598) that have been known to well-describe the functional protein changes, such as differential bitterness intensity^29^. Therefore, the diplotype for *TAS2R38* was computed using those three SNPs. Finally, 2 loci of LD block 1 (*TAS2R3/4*) and 3 loci of LD block 2 (*TAS2R38*) in chromosome 7 and 2 loci of LD block in chromosome 12 (*TAS2R50/48*) were applied for diplotype computation (Table 2).

**Table 2:** Descriptive data for selected/tagging single-nucleotide polymorphisms analysed in the current study.

Chr	Associated/ nearest gene	SNP	Obs HET	Pred HET	HW p-val	MAF	Alleles^a^	SNP type	LD block	Tagging SNP
7	*TAS2R3*	rs2270009	0.43	0.44	0.824	0.32	T:C	synonymous	1	Ch7B1_1
	*TAS2R4*	rs2234001	0.36	0.38	0.273	0.26	C:G	Val96Leu	1	Ch7B1_2
	*TAS2R38*	rs10246939	0.47	0.49	0.418	0.42	C:T	Ile296Val		Ch7B2_1
		rs1726866	0.47	0.49	0.455	0.43	G:A	Val262Ala	2	.
		rs713598	0.47	0.49	0.455	0.43	G:C	Ala49Pro		.
12	*TAS2R50*	rs10772397	0.34	0.34	0.818	0.22	T:C	synonymous	1	Ch12_1
	*TAS2R48*	rs1868769	0.15	0.15	0.913	0.08	A:G	synonymous	1	Ch12_2

Chr, chromosome; SNP, single-nucleotide polymorphism; Obs HET, observed heterozygosity; Pred HET, predicted heterozygosity; HW p-val, Hardy-Weinberg equilibrium test *p*-value; MAF, minor allele frequency; LD, linkage disequilibrium. ^a^Major; minor allele.

As a result of diplotype analyses, 6 and 4 types of diplotype were computed to exist in *TAS2R3/4* and *TAS2R38* LD blocks, respectively. Nine differential diplotypes were also present in the LD block of chromosome 12. The logistic regression models were only established with diplotypes with 3% or more of subjects to prevent false-positive findings due to a small number of subjects (Table 3). The findings suggested that subjects with the CC/TC *TAS2R3/4* diplotype were less likely to have PTC than those with TC/TC, the most frequent diplotype [odds ratio (OR) = 0.43, 95% confidence interval (95% CI): 0.23–0.79]. Furthermore, when the subjects were stratified based on the presence of CC haplotype, the CC haplotype reduced the risk for PTC compared with that of those lacking the CC haplotype (OR = 0.59, 95% CI: 0.36–0.97). However, other *TAS2R* diplotypes were not significantly associated with PTC susceptibility. Finally, the prognostic ability of *TAS2R* genetic variation in disease recurrence was analysed using the PTC stage and AGES (age, grade, extent of disease, size), MACIS (distant metastasis, age, complete surgical resection, invasion, size) and AMES (age, metastasis, extent of disease, size) criteria. However, those genetic variations did not influence the severity of PTC (Supplementary Tables S2–4).

**Table 3:** Distribution of *TAS2R* genetic variants and their association with risk for papillary thyroid carcinoma.

	Controls (%)	Case (%)	Odds ratio (95% CI)^a^	P
***TAS2R3/4*** **diplotype**				
TC/TC	229 (44.6)	121 (48.4)	1.00 (Reference)	
TC/CG	170 (33.1)	84 (33.6)	0.89 (0.64–1.27)	0.549
CC/TC	63 (12.3)	14 (5.60)	0.43 (0.23–0.79)	0.007
CG/CG	35 (6.82)	21 (8.40)	1.07 (0.59–1.94)	0.822
CC/CG	14 (2.73)	10 (4.00)	—	
CC/CC	2 (0.39)	—	—	
*/*	434 (84.6)	226 (90.4)	1.00 (Reference)	
CC/*	49 (15.4)	24 (9.60)	0.59 (0.36–0.97)	0.036
***TAS2R38*** **diplotype**				
PAV/PAV	171 (33.3)	87 (34.8)	1.00 (Reference)	
PAV/AVI	252 (49.1)	109 (43.6)	0.82 (0.58–1.17)	0.273
AVI/AVI	89 (17.4)	54 (21.6)	1.26 (0.82–1.95)	0.289
AVI/AAV	1 (0.19)	—		
***TAS2R*** **diplotype in chromosome 12** ^**b**^				
TA/TA	264 (51.7)	128 (52.0)	1.00 (Reference)	
CA/TA	148 (28.9)	69 (28.1)	0.97 (0.68–1.41)	0.938
TA/TG	48 (9.39)	25 (10.2)	1.09 (0.64–1.85)	0.761
CA/TG	26 (5.09)	10 (4.07)	0.87 (0.40–1.89)	0.733
CA/CA	21 (4.11)	10 (4.07)	0.88 (0.39–1.98)	0.751
CA/CG	3 (0.59)	1 (0.41)	—	—
CG/TG	1 (0.20)	1 (0.41)	—	—
CG/CG	—	1 (0.41)	—	—
TG/TG	—	1 (0.41)	—	—

95% CI, 95% confidence interval. Subjects with diplotypes with a frequency below 3% were excluded from the logistic regression tests based on rarity. ^a^The odds ratio was adjusted for the family history of thyroid cancer. ^b^Six individuals were excluded due to missing genotype data.

### Associations between *TAS2R* diplotype and biomarkers of thyroid function

No significant differences in the concentrations of TT3, FT4, TSH and Tg were noted between PTC phenotypes (Supplementary Table S5). However, when those biomarkers were analysed with *TAS2R* genetic characteristics, the *TAS2R3/4* diplotype exhibited a distinctive difference in TT3 concentration (Table 4). In the entire study population, the TT3 level for individuals with the CC/TC diplotype was lower than that for individuals with other diplotypes (p = 0.05). This trend towards TT3 and genotype was more clearly evident when the subjects were re-grouped, taking account of the presence of the CC haplotype (CC/*): CC haplotype carriers exhibited significantly lower levels of TT3 than non-carriers (1.04 ± 0.03 and 1.16 ± 0.01, p = 0.005). Further analyses taking account of the presence of the PTC phenotype also supported the association of the CC haplotype and TT3. In both the control and the PTC groups, the level of TT3 for CC haplotype carriers was lower compared with that of CC haplotype non-carriers (1.02 ± 0.04 and 1.14 ± 0.02, p = 0.022), although the difference in PTC cases was not statistically significant (p = 0.111). None of the genetic groups for the *TAS2R38* and *TAS2R50/48* diplotype exhibited meaningful associations with biomarkers related to thyroid function in all subjects as well as in cases and controls, respectively (Tables 5 and 6).

**Table 4:** Levels of biomarkers of thyroid function, taking *TAS2R3/4* diplotype and papillary thyroid carcinoma phenotype into account.

	TT3 (ng/mL)		FT4 (ng/dL)		TSH (μIU/mL)		Tg (ng/mL)	
	N	Mean (SE)	N	Mean (SE)	N	Mean (SE)	N	Mean (SE)
**All subjects**								
TC/TC	95	1.13 (0.02)	95	1.31 (0.02)	95	2.13 (0.13)	55	24.98 (7.36)
TC/CG	77	1.17 (0.02)	77	1.30 (0.02)	77	2.81 (0.28)	47	14.92 (2.97)
CC/TC	17	1.04 (0.03)	17	1.29 (0.05)	17	2.30 (0.29)	7	20.50 (3.75)
CG/CG	15	1.19 (0.05)	15	1.26 (0.04)	15	2.69 (0.63)	8	17.50 (3.66)
CC/CG	6	1.14 (0.05)	6	1.26 (0.09)	6	2.02 (0.68)	1	4.75
CC/CC	1	0.70	1	1.47	1	1.14	—	—
P^a^		0.05		0.613		0.652		0.24
*/*	187	1.16 (0.01)	187	1.30 (0.01)	187	2.45 (0.14)	110	20.14 (3.91)
CC/**	23	1.04 (0.03)	24	1.29 (0.04)	24	2.18 (0.26)	8	18.53 (3.80)
P^b^		0.005		0.803		0.536		0.364
**Controls**								
TC/TC	50	1.12 (0.02)	50	1.28 (0.03)	50	2.40 (0.20)	25	19.01 (4.39)
TC/CG	40	1.17 (0.03)	40	1.29 (0.02)	40	2.59 (0.33)	25	12.19 (2.04)
CC/TC	8	1.03 (0.04)	8	1.28 (0.06)	8	2.88 (0.44)	4	25.47 (3.84)
CG/CG	8	1.15 (0.08)	8	1.18 (0.07)	8	3.65 (1.16)	4	23.33 (3.66)
CC/CG	3	1.10 (0.06)	3	1.21 (0.10)	3	1.80 (0.79)	1	4.75
CC/CC	1	0.70	1	1.47	1	1.14	—	—
P^a^		0.159		0.327		0.496		0.084
*/*	98	1.14 (0.02)	98	1.27 (0.02)	98	2.57 (0.19)	55	16.30 (2.26)
CC/**	12	1.02 (0.04)	12	1.28 (0.05)	12	2.47 (0.38)	5	21.32 (5.10)
P^b^		0.022		0.996		0.974		0.317
**Cases**								
TC/TC	45	1.15 (0.03)	45	1.34 (0.03)	45	1.84 (0.15)	30	29.95 (13.02)
TC/CG	37	1.18 (0.03)	37	1.31 (0.03)	37	3.04 (0.45)	22	18.04 (5.93)
CC/TC	9	1.04 (0.06)	9	1.30 (0.07)	9	1.78 (0.32)	3	13.87 (5.51)
CG/CG	7	1.24 (0.07)	7	1.31 (0.05)	7	1.73 (0.21)	3	7.77 (2.13)
CC/CG	3	1.20 (0.10)	3	1.34 (0.20)	3	2.24 (1.28)	—	—
P^a^		0.159		0.651		0.179		0.408
*/*	89	1.17 (0.02)	89	1.32 (0.02)	89	2.33 (0.21)	55	23.98 (7.49)
CC/**	11	1.07 (0.25)	12	1.30 (0.06)	12	1.90 (0.36)	3	13.87 (5.51)
P^b^		0.111		0.739		0.415		0.933

TT3, total triiodothyronine; FT4, free thyroxine; TSH, thyroid-stimulating hormone; Tg, thyroglobulin, analysed only for anti-thyroglobulin antibody-negative subjects; N, number of subjects; SE, standard error. ^a^P-values come from the generalized linear models adjusted for family history of thyroid cancer (diplotype groups with fewer than 5 subjects were excluded from the comparison because of the rarity). ^b^P-values come from Student’s t-tests between diplotype groups (diplotypes with the CC haplotype versus all other diplotypes).

**Table 5:** Levels of biomarkers of thyroid function, taking *TAS2R38* diplotype and papillary thyroid carcinoma phenotype into account.

	TT3 (ng/mL)		FT4 (ng/dL)		TSH (μIU/mL)		Tg (ng/mL)	
	N	Mean (SE)	N	Mean (SE)	N	Mean (SE)	N	Mean (SE)
**All subjects**								
PAV/PAV	78	1.14 (0.02)	78	1.29 (0.02)	78	2.56 (0.23)	52	16.13 (2.71)
PAV/AVI	102	1.16 (0.02)	102	1.29 (0.02)	102	2.37 (0.18)	48	27.30 (8.36)
AVI/AVI	31	1.10 (0.03)	31	1.32 (0.04)	31	2.22 (0.30)	18	11.90 (2.74)
P^a^		0.441		0.795		0.658		0.181
**Controls**								
PAV/PAV	36	1.12 (0.03)	36	1.27 (0.02)	36	2.52 (0.23)	23	17.32 (4.75)
PAV/AVI	59	1.14 (0.02)	59	1.27 (0.02)	59	2.67 (0.27)	25	17.70 (1.94)
AVI/AVI	15	1.09 (0.04)	15	1.31 (0.05)	15	2.17 (0.46)	12	13.52 (3.88)
P^a^		0.682		0.593		0.816		0.263
**Cases**								
PAV/PAV	42	1.16 (0.03)	42	1.31 (0.03)	42	2.60 (0.39)	29	15.18 (3.14)
PAV/AVI	43	1.18 (0.03)	43	1.33 (0.03)	43	1.97 (0.19)	23	37.74 (17.26)
AVI/AVI	16	1.12 (0.05)	16	1.32 (0.05)	16	2.27 (0.40)	6	8.67 (2.70)
P^a^		0.548		0.744		0.356		0.452

TT3, total triiodothyronine; FT4, free thyroxine; TSH, thyroid-stimulating hormone; Tg, thyroglobulin, analysed only for anti-thyroglobulin antibody-negative subjects; N, number of subjects; SE, standard error. ^a^P-values come from the generalized linear models adjusted for family history of thyroid cancer.

**Table 6:** Levels of biomarkers of thyroid function, taking *TAS2R50/48* diplotype on chromosome 12 and papillary thyroid carcinoma phenotype into account.

	TT3 (ng/mL)		FT4 (ng/dL)		TSH (μIU/mL)		Tg (ng/mL)	
	N	Mean (SE)	N	Mean (SE)	N	Mean (SE)	N	Mean (SE)
**All subjects**								
TA/TA	106	1.13 (0.02)	106	1.30 (0.02)	106	2.38 (0.15)	59	21.97 (6.85)
CA/TA	60	1.14 (0.03)	60	1.31 (0.02)	60	2.55 (0.32)	36	20.21 (3.94)
TA/TG	27	1.19 (0.04)	27	1.29 (0.03)	27	1.96 (0.26)	11	12.91 (4.07)
CA/TG	11	1.12 (0.04)	11	1.29 (0.04)	11	2.52 (0.61)	8	17.25 (3.11)
CA/CA	5	1.12 (0.06)	5	1.25 (0.04)	5	2.94 (0.64)	4	14.93 (6.01)
CA/CG	1	1.10	1	1.08	1	6.59	—	—
CG/TG	1	1.10	1	1.21	1	4.45	—	—
P^a^		0.723		0.977		0.577		0.477
**Controls**								
TA/TA	52	1.12 (0.02)	52	1.26 (0.02)	52	2.49 (0.23)	29	15.22 (1.72)
CA/TA	32	1.14 (0.03)	32	1.29 (0.03)	32	2.65 (0.36)	18	20.40 (6.09)
TA/TG	15	1.13 (0.05)	15	1.30 (0.04)	15	1.97 (0.41)	5	15.75 (8.25)
CA/TG	8	1.14 (0.04)	8	1.26 (0.05)	8	3.21 (0.69)	6	14.89 (3.22)
CA/CA	2	1.15 (0.15)	2	1.29 (0.01)	2	2.53 (1.44)	2	13.25 (5.12)
CA/CG	1	1.10	1	1.08	1	6.59	—	—
P^a^		0.899		0.751		0.179		0.809
**Cases**								
TA/TA	54	1.15 (0.02)	54	1.33 (0.02)	54	2.27 (0.19)	30	28.50 (13.4)
CA/TA	28	1.15 (0.04)	28	1.34 (0.04)	28	2.42 (0.56)	18	20.01 (5.18)
TA/TG	12	1.27 (0.06)	12	1.27 (0.05)	12	1.95 (0.31)	6	10.55 (3.61)
CA/TG	3	1.07 (0.07)	3	1.37 (0.06)	3	0.70 (0.23)	2	24.34 (7.00)
CA/CA	3	1.10 (0.06)	3	1.21 (0.06)	3	3.22 (0.76)	2	16.60 (13.6)
CG/TG	1	1.10	1	1.21	1	4.45	—	—
P^a^		0.133		0.629		0.599		0.758

TT3, total triiodothyronine; FT4, free thyroxine; TSH, thyroid-stimulating hormone; Tg, thyroglobulin, analysed only for anti-thyroglobulin antibody-negative subjects; N, number of subjects; SE, standard error. ^a^P-values come from the generalized linear models adjusted for family history of thyroid cancer (diplotype groups with fewer than 5 subjects were excluded from the comparison because of rarity).

## Discussion

This study hypothesized that T2R bitterness receptors may modify susceptibility to PTC and that the T2R-PTC association may be associated with genetically mediated thyroid function. Here, the current findings supported this idea: variations in the *TAS2R3* and *TAS2R4* genes reduced the risk of PTC, and the differential concentration of TT3 modified by those *TAS2R* genetic variations may be associated with the potential underlying mechanism of PTC development and progression.

Earlier studies of T2Rs mainly focused on their role in bitterness sensing. However, the presence of extra-orally expressed T2R has been reported and suggests that T2Rs play differential roles beyond bitterness perception that are tailored to various locations^30,31^. To date, growing evidence suggests that T2R taste receptors possess regulatory effects in human metabolism and disease. The physiological roles of those taste receptors, including innate immunity, secretion, contraction and relaxation of smooth muscle cells, have been conjectured to explain such pathological roles of T2R in disease aetiology^31,32^; however, most of the mechanisms were only presumptive. A study by Clark *et al*. confirmed the expression of T2R on thyrocytes and their mechanism of action^24^. The study verified that T2R agonists inhibited the intracellular levels of calcium and iodine ions, which regulate the production of thyroid hormones, only in the presence of TSH. This finding could provide evidence regarding how T2Rs and their genetic variations influence energy metabolism and body composition. Furthermore, these findings may also imply a defence mechanism employed by the thyroid against noxious molecules^24^. Many phytotoxins and harmful compounds possess bitterness characteristics^23,33^. The chemosensing of pernicious bitterness ex-/endogenous molecules in the thyroid may trigger the protective mechanism via functional alterations of thyrocytes, such as the inhibition of hormone production^24^. Consistent with this finding, our results also suggest that the variant T2R proteins might lead to differential efficacy in chemosensing and subsequent metabolic alterations, hence reducing the risk for PTC.

The effect-modifying haplotype *TAS2R3/4* CC consists of two genetic variations: the minor allele C of rs2270009 in *TAS2R3* and the major allele C of rs2234001 in *TAS2R4*. *TAS2R3* and *TAS2R4* reside closely on chromosome 7. These receptors respond to multiple bitterness substances, including chloroquine, propylthiouracil and denatonium benzoate^23^. Notably, the C to T change at *TAS2R3* rs2270009 is a silent change; this sequence alteration does not lead to an amino acid alteration. However, numerous studies have suggested that synonymous changes lead to subsequent alterations in mRNA splicing and protein folding, ultimately modifying enzyme function^34,35^. Expression quantitative trait loci database analyses also provide supportive data suggesting that *TAS2R3* rs2270009 altered 5 transcription factor binding motifs (E2F, NF-Y, Pax-4, CEBPD, and Pbx-1) and caused decisive changes in the expression of other genes in thyroid tissue (Supplementary Tables S6 and S7)^36,37^. In contrast, although molecular modelling studies have been revealing the structure-functional influence of genetic variations in *TAS2R4*, thus far, little is known for V96L, rs2234001^20^. As epidemiological evidence, current findings suggest that the alterations in the secondary structure and stability of T2R3 with the concomitant expression of rs2234001 C–T2R4 may cause altered ligand sensing, mediating the protective effect against PTC and differential TT3 levels. The potential changes in the expression of other genes, including *WEE2* and *WEE2-AS1*, linked with these genetic variations may also contribute to such alteration of the maturation and/or function of thyrocytes and, further, the risk for PTC (Supplementary Table S7)^36,38^. Additionally, the expression of T2R38 on thyrocytes was evident^24^, but *TAS2R38* taste receptor genetic variation was not associated with any thyroid-related variables examined, the risk for PTC or biomarkers. Although *TAS2R38* is the major target of taste receptor sequence variation studies, controversies exist. Studies have reported that *TAS2R38* variations showed minimal effects on food intake^13,15^ or disease susceptibility^39–41^. Furthermore, other T2R proteins were observed to play tissue-specific physiological roles, and the biochemical and pharmacological characteristics of their variants for responding molecules have been verified^20^. For better understanding and health/therapeutic applications of bitterness chemosensing protein, more comprehensive approaches are required, including diverse T2Rs and genetic variants; current findings could be referenced in future structure-function studies.

To obtain more precise evidence regarding whether the association between *TAS2R* genetic variations and reduced PTC risk is associated with thyroid functional changes, we examined the levels of four thyroid function-related markers. The analyses showed that the *TAS2R3/4* CC haplotype was associated with TT3 concentration. Overall, CC haplotype carriers, who were at the lower risk for PTC, exhibited lower TT3 concentrations than non-carriers. This trend of TT3 concentration and the haplotype were also retained when PTC phenotype was taken into account. In each phenotype group, CC haplotype carriers showed lower levels of TT3 than non-carriers. Furthermore, the TT3 levels of CC haplotype carriers without PTC were the lowest, while those of non-carrier PTC cases tended to be highest. Although controversial, studies have reported that the T3 level is a prediction marker or risk-modifying factor in disease aetiology. Elevated TT3 levels are positively associated with the risk for breast and renal cancer^42–44^. In the current study, TT3 levels in PTC cases also tended to be higher than controls, although the difference was not statistically significant (Supplementary Table S5). The precise mechanism for such T3-induced cancer risk has not yet been clearly demonstrated. However, one study suggested a new molecular mechanism^45^: T3 enhanced TRb1/Oct-1-mediated cyclin D1 transcription, which further promoted PTC cell proliferation. Given these findings, here, we carefully describe a potential association with variant T2R proteins, thyroid function and PTC susceptibility. The structural alteration of the *TAS2R3/4* CC haplotype could lead to a change in agonist sensing of T2R3 and T2R4 receptors. The altered efficacy of molecular sensing of variant receptors may regulate the function of thyrocytes and their reduced activity and/or T3 thyroid hormone levels, which may act protectively against potential PTC carcinogenic mechanisms. This hypothesis regarding the association between *TAS2R3/4* variation and TT3 levels may explain the findings of differential risk for PTC based on *TAS2R3/4* genotype, at least in part.

T3 and T4 are both produced in thyrocytes. T3 is biologically active, but the direct production of T3 in the thyroid is only responsible for 20% of T3; the remaining 80% is produced by conversion from T4^46,47^. In the study of Clark *et al*. in an Amish population^24^, *TAS2R42* rs5020531 regulated free T3 and T4 levels, and FT4 exhibited a greater association with that genetic factor. However, in these Korean females, the *TAS2R* diplotype, including *TAS2R42*, did not exhibit a distinctive influence on examined variables (the analyses with rs5020531 did not reveal significant effects on TT3 and FT4 levels; data not shown). The *TAS2R3/4* diplotype exclusively exhibited an association with TT3 concentration but not FT4. Some hypotheses could be suggested to explain the discrepancies between these studies and gene-hormone associations. First, the Amish are a Caucasian and rural-living population that possess significant differences in genetic and milieu factors from Korean females^48^. Such differential ethnic characteristics could contribute to the differential distribution of LD patterns and tagging SNPs and, ultimately, the genetic variation-hormone association. Second, the differential study design (case-control versus cohort study) may have resulted in discordance between findings. Finally, unlike T2R42, T2R3/4 proteins may regulate only the T3 produced directly in thyrocytes. The thyrocyte-expressed T2R3/4 proteins may selectively affect the production and/or transportation of T3 across the follicular lumen, cell and endothelium and its release into the blood. Additionally, T2R3/4 proteins may also be involved in the conversion of T4 into T3 in each target tissue. T4 bound to plasma protein is transported to the target organ and converted into T3 by deiodinase enzymes. Tissue-specific T2R3/4 bitterness receptors may be related with such a deiodination process, hence leading to differences in TT3 concentrations. However, the TT3 concentration could be modified due to various reasons. The TT3 level is also essentially regulated by the negative feedback mechanism. Lower T3 and T4 levels initiate the production and release of TSH, therefore maintaining the T3 and T4 concentrations. However, in the current study, *TAS2R3/4* genetic variations did not modify the level of TSH, only influencing the TT3 concentration. We could not dismiss the potential that *TAS2R3/4* genetic variation may be associated with the activity or function of T3 rather than its expression level, but the evidence is limited. The present analyses of hormones and *TAS2R* variants were only performed in relatively small numbers of subjects, and the findings and hypotheses should be confirmed in a large cohort study and will require mechanistic evidence from experimental models.

The present study provides preliminary epidemiological evidence that genetic variations in *TAS2Rs* are associated with PTC risk and thyroid function. The subjects with homogeneous characteristics and the analyses of pre-diagnostic levels of biomarkers are the strengths of the study; yet, the work may harbour limitations. First, the study was performed in a relatively small population. The findings have not yet been confirmed in secondary or experimental studies, and the potential effects of multiple statistical tests were not adjusted. Second, the LD patterns, tagging SNPs and study findings could vary depending on the study population. Analyses with additional genetic data and/or in other ethnicities may lead to differential results compared with the current findings. Third, due to limited resources, the measurements of thyroid function and autoimmunity markers were performed only in a selected group of 110 cases and 101 controls. These subjects were only chosen based on the availability of pre-diagnostic serum samples, and unknown bias may exist. Lastly, study subjects were recruited from the Cancer Screening Cohort Study (CSCS), a major cancer study cohort in Korea that has a relatively short history. The mean follow-up time for those PTC cases with pre-diagnostic samples was 3.15 ± 2.79 years; therefore, the biomarkers may lack power as prognostic indicators. For these reasons, the study’s findings should be interpreted with caution.

In conclusion, genetic variations in bitterness receptor *TA2Rs* may modify susceptibility to PTC in Korean females, and genetic variation-mediated thyroid function may be associated with PTC carcinogenesis. Given the metabolic and therapeutic importance of thyroid and T2R GPCR, the current findings add knowledge regarding chemosensing mechanisms and disease treatment.

## Methods

### Study population and data collection

**Figure 2:**
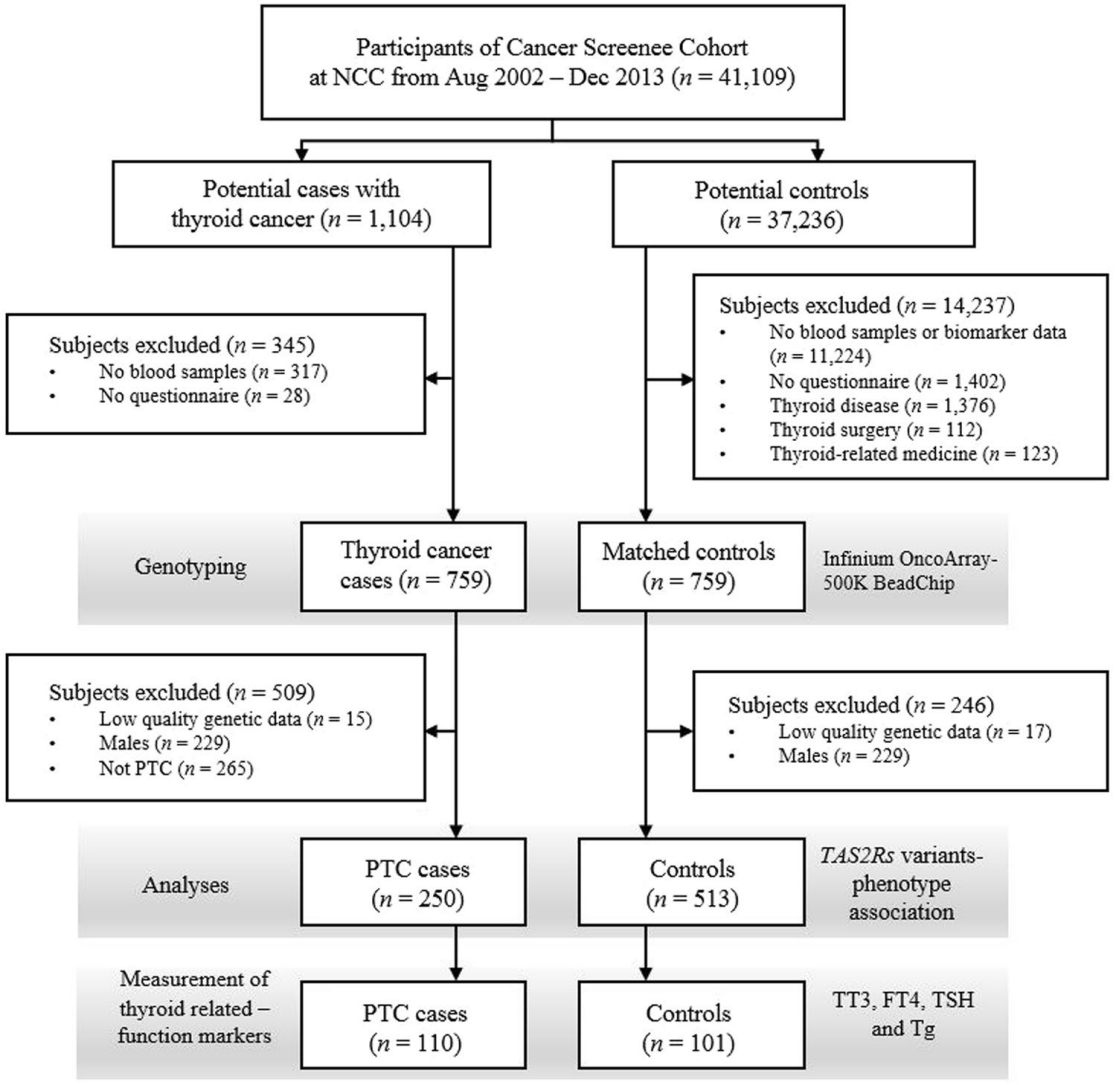
A simplified flowchart of the current study. NCC, National Cancer Center; PTC, papillary thyroid carcinoma; TT3, total triiodothyronine; FT4, thyroxine; TSH, thyroid-stimulating hormone; Tg, thyroglobulin.

The subjects of this case-control study were recruited from among individuals who visited the National Cancer Center, Korea, between August 2002 and December 2013. The details of the study population have been described previously (Fig. 2)^49,50^. Briefly, a total of 41,109 volunteers older than 30 years of age who underwent health-screening examinations (a benefit programme of the National Health Insurance) were participants in an ongoing CSCS. At the baseline evaluation, those individuals were requested to complete a self-administered questionnaire with information on socio-demographic characteristics, personal and family medical histories and reproductive and lifestyle factors. Blood samples were also collected at this point. Using the linkage with the Korea Central Cancer Registry database (ICD10 code C73), 1,104 patients from the CSCS were identified as having thyroid cancer, and 37,236 were defined as potential controls. Among these potential cases and controls, 759 thyroid cases and 759 age- (CSCS entry) and sex-matched controls were genotyped. Then, 250 female PTC cases defined by endocrinologists and 513 female controls were subjected to analyses of the association between *TAS2R* variations and phenotype. The prognostic effect of genetic variations was also estimated using the carcinoma stage and AGES, MACIS and AMES classification systems. The PTC stage and risk prediction score/group were defined following the American Thyroid Association classification system (7th edition)^51^. Among those subjects, the levels of four thyroid-related functional markers were determined in 110 PTC cases and 101 controls from whom we were able to obtain pre-diagnostic serum samples.

### Measurement of biomarkers

The serum samples collected at the baseline evaluation were stored at −196 °C until analyses. Electrochemiluminescence immunoassays (ELCLIA; Molecular Analytics E170, Roche kit, Roche, Mannheim, Germany) were applied for the measurements. The techniques were detailed previously^1^ and reference range for the biomarkers are presented in Supplementary Table S5.

### Genetic data production and analyses

Genomic DNA was extracted using the following commercial kit and instrument: MagAttract DNA Blood M48 Kit (Qiagen, Hilden, Germany) and BioRobot M48 automatic extraction equipment (Qiagen, Hilden, Germany). Genomic DNA samples were stored at −80 °C until the analyses. *TAS2R* genotypes were determined using 500 ng of genomic DNA and an Infinium OncoArray-500K BeadChip (Illumina Inc., CA, USA). For quality control (QC) of the produced genotypes, loci exhibiting call rates <95%, minor allele frequencies <0.01 and deviation from Hardy-Weinberg equilibrium (p < 1 × 10^−6^) were eliminated. Strand alignment and phasing were performed using PLINK v1.07^52^ and SHAPEIT2^53^. Thereafter, imputation was performed with IMPUTE2 software^54^, referencing the 1000 Genomes EAS Phase III reference panel (integrated variant set release in NCBI build 37, hg19). For QC of the imputed results, only the variants with info scores >0.6 were included in subsequent evaluations. The haplotype and tagging SNPs were evaluated using Haploview^28^, and the diplotype was computed with FAMHAP^55^. The functional alterations of T2R variant proteins were estimated using HaploReg v4.1^37^.

### Statistical analyses

To compare the general characteristics of the study subjects depending on PTC phenotype, chi-squared and Student’s t-tests were used. ORs and 95% CIs were estimated to predict the association between *TAS2R* genetic variation and PTC risk using logistic regression models. Analysis of variance (ANOVA) was performed to assess the differences in biomarkers between PTC phenotypes or *TAS2R* diplotypes. The chi-squared tests and ANOVA were also applied to test the association between *TAS2R* genetic variation and PTC stage as well as various risk prediction criteria and groups. Those logistic regression and ANOVA models were established in the presence of covariates. For *post hoc* analyses, Tukey’s test was used. All continuous variables were applied in the statistical model after log-transformation. All statistical analyses were conducted using SAS 9.3 software (SAS Institute Inc., Cary, NC, USA), and a two-sided p-value < 0.05 was considered significant.

### Ethics statement

Ethical approval for this study was obtained from the Institutional Review Board of the NCC (#NCC2016-0088). All actual study procedures were conducted following the approved protocol, and informed consent was obtained from participants prior to study commencement.

## Electronic supplementary material

Supplementary materials

## Data Availability

All datasets generated during the present study are available from the corresponding author on reasonable request.
