# Supplementary material for: Genetic variations in TAS2R3 and TAS2R4 bitterness receptors modify papillary carcinoma risk and thyroid function in Korean females
Source: Sci Rep. 2018 Oct 9;8:15004. doi: 10.1038/s41598-018-33338-6 (PMC6177438; doi:10.1038/s41598-018-33338-6)
Supplement: Supplementary file 1 — Supplementary materials [file 41598_2018_33338_MOESM1_ESM.pdf]

Supplementary materials

Genetic variations in *TAS2R3* and *TAS2R4* bitterness receptors modify papillary carcinoma risk and thyroid function in Korean females

Author names and affiliations:

Jeong-Hwa Choi[1,2], Jeonghee Lee[1], Sarah Yang[1,3], Eun Kyung Lee[4], Yul Hwangbo[4], Jeongseon Kim[1*]

[1] Department of Cancer Biomedical Science, Graduate School of Cancer Science and Policy, National Cancer Center, 323 Ilsan-ro, Ilsandong-gu, Goyang-si, Gyeonggi-do, 10408, Korea.

[2] Department of Food Science and Nutrition, Keimyung University, 1095, Dalgubeol-daero, Dalseo-gu, Daegu, 42601, Korea

[3]Complex Disease & Genome Epidemiology Branch, Department of Epidemiology, School of Public Health, Seoul National University, 1 Gwanak-ro, Gwanak-gu, Seoul, 08826, Korea.

[4]Center for Thyroid Cancer, National Cancer Center Hospital, National Cancer Center, 323 Ilsan-ro, Ilsandong-gu, Goyang-si, Gyeonggi-do, 10408, Korea

***Correspondence:** Jeongseon Kim, E-mail: jskim@ncc.re.kr

Supplementary Table S1. Descriptive data of single-nucleotide polymorphisms analysed in the current study.

chr, chromosome; SNP, single-nucleotide polymorphism; Obs HET, observed heterozygosity; Pred HET, predicted heterozygosity; HW p-val, Hardy-Weinberg equilibrium test *p*-value; MAF, minor allele frequency; LD, linkage disequilibrium.
[a] major; minor allele. [b] near the 5' end of a gene. [c] near the 3' end of a gene.

chr	Associated/ nearest gene	SNP	position	Obs HET	Pred HET	HW pval	MAF	Alleles[a]	SNP type	LD block	Tagger
7	*TAS2R16*	rs860170	122635024	0.495	0.486	0.675	0.417	T:C	Arg222His		
	TAS2R3	rs2270009	141464765	0.434	0.438	0.824	0.324	T:C	synonymous	1	Ch7B1_1
		rs76494765	141464810	0.088	0.084	0.439	0.044	C:T	synonymous		
	TAS2R4	rs2233996	141478297	0.050	0.049	1.000	0.025	G:C	synonymous		
		rs2233998	141478308	0.434	0.438	0.824	0.324	C:T	Phe7Ser	1	
		rs2234001	141478574	0.364	0.381	0.273	0.256	C:G	Val96Leu	1	Ch7B1_2
		rs2234002	141478800	0.366	0.381	0.296	0.256	A:G	Ser171Asn	1	
	TAS2R38	rs10246939	141672604	0.473	0.488	0.418	0.424	C:T	Ile296Val	2	Ch7B2_1
		rs1726866	141672705	0.474	0.489	0.455	0.425	G:A	Val262Ala	2	
		rs145970530	141673074	0.034	0.034	1.000	0.017	T:C	Lys139Arg		
		rs713598	141673345	0.474	0.489	0.455	0.425	G:C	Ala49Pro	2	
		rs140737317	141674613	0.029	0.028	1.000	0.014	A:G	nearGene-5		
	TAS2R40	rs10260248	142919731	0.023	0.023	1.000	0.012	C:A	Ser187Tyr		
	TAS2R60	rs4595035	143141475	0.169	0.168	1.000	0.093	C:T	synonymous		
12	*TAS2R50*	rs10772397	11138683	0.338	0.342	0.818	0.219	T:C	synonymous	1	Ch12B1_1
		rs1376251	11138852	0.338	0.342	0.818	0.219	T:C	Cys203Tyr	1	
	TAS2R49	rs10845279	11149711	0.337	0.344	0.611	0.221	A:C	Arg255Leu	1	
		rs10845280	11149720	0.337	0.344	0.611	0.221	G:A	Phe252Ser	1	
		rs10845281	11149769	0.337	0.344	0.611	0.221	C:T	Ile236Val	1	
		rs12226919	11150033	0.337	0.344	0.611	0.221	T:G	His148Asn	1	
		rs12226920	11150046	0.337	0.344	0.611	0.221	T:G	His143Gln	1	
		rs11054142	11150214	0.337	0.344	0.611	0.221	A:G	synonymous	1	
		rs7135018	11150240	0.318	0.326	0.575	0.205	T:C	Lys79Glu	1	
		rs11054143	11150319	0.337	0.344	0.611	0.221	C:T	synonymous	1	
	TAS2R48	rs10772420	11174276	0.318	0.326	0.575	0.205	G:A	Arg299Cys	1	
		rs1868769	11174753	0.149	0.147	0.913	0.080	A:G	synonymous	1	Ch12B1_2
		rs12313469	11175087	0.318	0.326	0.575	0.205	G:A	synonymous	1	
	TAS2R45	rs10772423	11183217	0.318	0.326	0.575	0.205	C:T	Val240Ile	1	
		rs10845293	11183255	0.318	0.326	0.575	0.205	G:A	Ala227Val	1	
		rs12370363	11183512	0.318	0.326	0.575	0.205	A:G	synonymous	1	
		rs10845295	11183832	0.318	0.326	0.575	0.205	G:A	Arg35Trp	1	
	TAS2R46	rs2708381	11214145	0.316	0.322	0.653	0.202	C:T	STOP-GAIN	1	
		rs2708380	11214212	0.316	0.322	0.653	0.202	A:T	Leu228Met	1	
		rs202165583	11214633	0.028	0.030	0.310	0.015	G:C	synonymous		
		rs201585352	11214634	0.028	0.030	0.310	0.015	A:C	Val87Gly		
	TAS2R47	rs2599404	11286088	0.319	0.324	0.762	0.203	A:C	Non-coding	1	
		rs2600355	11286790	0.319	0.324	0.762	0.203	A:C	Non-coding	1	

(Table continued)

12	*TAS2R42*	rs1451766	11333632	0.341	0.344	0.879	0.220	T:A	Non-coding	1	
		rs319271	11333633	0.323	0.326	0.883	0.205	T:G	Non-coding	1	
		rs319272	11334369	0.323	0.326	0.883	0.205	C:A	Non-coding	1	
		rs319273	11334853	0.323	0.326	0.848	0.205	T:C	Non-coding	1	
		rs319274	11336273	0.323	0.326	0.883	0.205	T:G	Non-coding	1	
		rs319276	11336681	0.028	0.030	0.310	0.015	A:C	Non-coding		
		rs35650731	11337371	0.341	0.344	0.879	0.220	C:T	Non-coding	1	
		rs319277	11337442	0.028	0.030	0.310	0.015	A:G	nearGene-3[b]		
		rs1669409	11338555	0.028	0.030	0.310	0.015	T:A	nearGene-3	1	
		rs1669410	11338559	0.325	0.327	0.941	0.206	C:T	nearGene-3	1	
		rs1817104	11338589	0.325	0.327	0.941	0.206	G:T	nearGene-3	1	
		rs1650017	11338613	0.028	0.030	0.310	0.015	G:C	Pro311Ala		
		rs1669411	11338614	0.028	0.030	0.310	0.015	G:A	synonymous		
		rs1669412	11338669	0.325	0.327	0.941	0.206	C:T	Gln292Arg	1	
		rs1451772	11338750	0.325	0.327	0.941	0.206	T:C	Cys265Tyr	1	
		rs1669413	11338781	0.028	0.030	0.310	0.015	A:C	Trp255Gly		
		rs5020531	11338957	0.343	0.344	0.937	0.221	G:A	Phe196Ser	1	
		rs1650019	11338983	0.028	0.030	0.310	0.015	C:T	synonymous		
		rs35969491	11339020	0.343	0.344	0.937	0.221	A:T	Tyr175Phe	1	
		rs34272839	11341118	0.343	0.344	0.937	0.221	T:C	nearGene-5[c]	1	
		rs1669414	11341169	0.325	0.327	0.941	0.206	T:A	nearGene-5	1	
		rs17817150	11341521	0.343	0.344	0.937	0.221	T:C	nearGene-5	1	
		rs80334300	11341877	0.026	0.028	0.285	0.014	T:A	Non-coding		
		rs61928650	11341878	0.028	0.030	0.310	0.015	C:T	Non-coding		
		rs1669415	11342401	0.028	0.030	0.310	0.015	T:C	Non-coding		
		rs1669416	11342415	0.028	0.030	0.310	0.015	C:G	Non-coding		
		rs1669417	11342525	0.028	0.030	0.310	0.015	A:G	Non-coding		
		rs35499317	11342582	0.343	0.344	0.937	0.221	C:T	Non-coding	1	
		rs1669418	11342751	0.325	0.327	0.941	0.206	G:A	Non-coding	1	
		rs1650020	11343326	0.325	0.327	0.941	0.206	G:A	Non-coding	1	
		rs1669419	11343422	0.028	0.030	0.310	0.015	A:G	Non-coding		
		rs1650021	11343964	0.028	0.030	0.310	0.015	A:T	Non-coding		
		rs7132674	11344304	0.342	0.344	0.928	0.221	G:A	Non-coding	1	
		rs6488358	11344381	0.342	0.344	0.928	0.221	T:G	Non-coding	1	
		rs7302711	11344630	0.342	0.344	0.928	0.221	T:C	Non-coding	1	
		rs1669420	11344847	0.028	0.030	0.310	0.015	G:A	Non-coding		

Supplementary Table S2. Distribution of *TAS2R* genetic variation and the papillary thyroid carcinoma stage.

PTC, papillary thyroid carcinoma. The data represent the number of subjects (%). The PTC stage was defined following the cancer staging manual from the American Joint Committee on Cancer (7th edition). Some cases (n=25) were not determined due to the lack/ambiguity of stage information. Subjects with diplotypes less than 3% were excluded from the tests due to their rarity. [a] P-values from the chi-squared tests between *TAS2R* genetic variations and the number of patients in each stage of PTC. [b] Four individuals were excluded due to missing genotype data.

	PTC stage				P[a]
	1	2	3	4a	
TAS2R3/4 diplotype					
TC/TC	68 (54.8)	-	35 (40.2)	5 (38.5)	0.2330
TC/CG	35 (28.2)	1 (100)	37 (42.5)	5 (38.5)	
CC/TC	6 (4.8)	-	3 (3.5)	2 (15.4)	
CG/CG	10 (8.1)	-	8 (9.2)	-	
CC/CG	5 (4.0)	-	4 (4.6)	1 (7.7)	
**/**	113 (91.1)	1 (100)	80 (91.9)	10 (76.9)	0.2132
CC/**	11 (8.9)	-	7 (8.1)	3 (23.1)	
TAS2R38 diplotype					
PAV/PAV	38 (30.7)	-	34 (39.1)	5 (38.5)	0.5903
PAV/AVI	62 (50.0)	-	34 (39.1)	5 (38.5)	
AVI/AVI	24 (19.3)	1 (100)	19 (21.8)	3 (23.1)	
TAS2Rs in chromosome 12[b]					
TA/TA	66 (54.6)	1 (100)	40 (46.5)	5 (38.5)	0.3935
CA/TA	30 (24.8)	-	29 (33.7)	4 (30.8)	
TA/TG	16 (13.2)	-	6 (6.9)	3 (23.1)	
CA/TG	3 (2.5)	-	5 (5.8)	1 (7.7)	
CA/CA	6 (4.9)	-	3 (3.5)	-	
CA/CG	-	-	1 (1.2)	-	
CG/TG	-	-	1 (1.2)	-	
TG/TG	-	-	1 (1.2)	-	

Supplementary Table S3. AGES and MACIS risk index of papillary thyroid carcinoma by *TAS2Rs* genetic variation.

AGES, age, grade, extent of disease, size; MACIS, distant metastasis, age, complete surgical resection, invasion, size. Data are presented as the mean (standard error). The risk prediction score/group was defined following the American Thyroid Association classification system (7th edition). Genotypes with less than 3% of individuals were excluded from statistical analysis due to their rarity. [a]P-values were obtained from generalized linear models adjusted with the family history of thyroid cancer.

	AGES	P[a]	MACIS	P[a]
TAS2R3/4 diplotype				
TC/TC	2.69 (0.11)	0.0609	4.59 (0.08)	0.0641
TC/CG	3.12 (0.11)		4.89 (0.10)	
CC/TC	3.17 (0.21)		4.73 (0.27)	
CG/CG	3.16 (0.12)		4.77 (0.14)	
CC/CG	3.26 (0.38)		5.08 (0.30)	
**/**	2.90 (0.08)	0.3096	4.72 (0.06)	0.4378
CC/**	3.21 (0.21)		4.90 (0.20)	
TAS2R38 diplotype				
PAV/PAV	3.0 (0.12)	0.4154	4.81 (0.10)	0.6639
PAV/AVI	2.82 (0.11)		4.67 (0.08)	
AVI/AVI	3.03 (0.14)		4.77 (0.11)	
TAS2Rs in chromosome 12[b]				
TA/TA	2.87 (0.10)	0.6169	4.70 (0.08)	0.4625
CA/TA	2.98 (0.14)		4.79 (0.12)	
TA/TG	2.89 (0.19)		4.66 (0.16)	
CA/TG	3.37 (0.17)		5.05 (0.19)	
CA/CA	2.47 (0.40)		4.36 (0.26)	
CA/CG	3.42		4.86	
CG/TG	3.67		5.26	
TG/TG	4.43		6.47	

Supplementary Table S4. AGES and AMES risk-group classification and *TAS2Rs* genetic variation.

AGES, age, grade, extent of disease, size; AMES, age, metastasis, extent of disease, size. Data are presented as the mean and standard error in the brackets. The risk prediction score/group was defined following the American Thyroid Association classification system (7th edition). Individuals with less than 3% of genotypes were excluded from statistical analysis due to their rarity. The MACIS risk group (7 high risk) was not analysed because only one case was in the high-risk group. [a] Subjects were defined as high-risk if the AGES score was higher than 4. [b]P-values were from generalized linear models adjusted with the family history of thyroid cancer.

	AGES risk group [a]		P^b	AMES risk group		P^b
	Low	High		Low	High	
TAS2R3/4 diplotype						
TC/TC	97 (48.5)	11 (44.0)	0.4275	58 (54.2)	50 (42.4)	0.2614
TC/CG	66 (33.0)	12 (48.0)		31 (28.9)	47 (39.8)	
CC/TC	10 (5.0)	1 (4.0)		5 (4.7)	6 (5.1)	
CG/CG	18 (9.0)	-		10 (9.4)	8 (6.8)	
CC/CG	9 (4.5)	1 (4.0)		3 (2.8)	7 (5.9)	
**/**	181 (90.5)	23 (92.0)	0.8079	99 (92.5)	105 (88.9)	0.3619
CC/**	19 (9.5)	2 (8.0)		8 (7.5)	13 (11.1)	
TAS2R38 diplotype						
PAV/PAV	67 (33.5)	10 (40.0)	0.8046	33 (30.9)	44 (37.3)	0.5175
PAV/AVI	91 (45.5)	10 (40.0)		52 (48.6)	49 (41.5)	
AVI/AVI	42 (21.0)	5 (20.0)		22 (20.6)	25 (21.2)	
TAS2Rs in chromosome 12[b]						
TA/TA	99 (50.5)	13 (52.0)	0.8305	53 (50.5)	59 (50.9)	0.9894
CA/TA	55 (28.1)	8 (32.0)		31 (29.5)	32 (27.6)	
TA/TG	24 (12.2)	1 (4.0)		12 (11.4)	13 (11.2)	
CA/TG	8 (4.1)	1 (4.0)		4 (3.8)	5 (4.1)	
CA/CA	8 (4.1)	1 (4.0)		5 (4.8)	4 (3.5)	
CA/CG	1 (0.5)	-		-	1 (0.9)	
CG/TG	1 (0.5)	-		-	1 (0.9)	
TG/TG	-	1 (4.0)		-	1 (0.9)	

Supplementary Table S5. Reference ranges and levels of biomarkers of thyroid function, according to papillary thyroid carcinoma phenotype.

TT3, Total triiodothyronine; FT4, Free thyroxine; TSH, Thyroid-stimulating hormone; Tg, Thyroglobulin. [a] p-values from Student's t-tests between papillary thyroid carcinoma phenotypes. [b] Tg was analysed only for anti-thyroglobulin antibody-negative subjects. Anti-thyroglobulin antibody was defined as negative if ≤115.0 IU/mL.

Mean (standard error)	Normal range	All subjects (*n*=211)	Controls (*n*=101)	Cases (*n*=110)	P[a]
TT3 (ng/mL)	0.82-2.00	1.14 (0.01)	1.13 (0.02)	1.16 (0.02)	0.1927
FT4 (ng/dL)	0.93-1.70	1.30 (0.01)	1.27 (0.02)	1.32 (0.02)	0.0531
TSH (μIU/mL)	0.27-4.20	2.42 (0.13)	2.56 (0.17)	2.28 (0.19)	0.1553
TG (ng/mL)[b]	1.40-78.0	20.03 (3.65)	16.72 (2.11)	23.45 (7.11)	0.3589

Supplementary Table S6. Regulatory motifs changed for *TAS2R3* rs2270009 and *TAS2R4* rs2234001 in HaploReg V4.1.

chr, chromosome; pos, position; Ref, reference; Alt, alteration; AFR, African; AMR, American; ASN, Asian; EUR, European; eQTL, expression quantitative trait loci

chr	pos (hg38)	variant	Ref allele	Alt allele	AFR freq	AMR freq	ASN freq	EUR freq	Motifs changed	Selected eQTL hits	GENCODE genes	dbSNP functional annotation
7	141764965	rs2270009	C	T	0.2	0.52	0.67	0.46	E2F, NF-Y, Pax-4, CEBPD, and Pbx-1	111 hits	TAS2R3	synonymous
7	141778774	rs2234001	G	C	0.34	0.56	0.75	0.47	RXRA, Smad3	116 hits	TAS2R4	missense

Supplementary Table S7. Thyroid tissue-specific expression quantitative trait loci (eQTL) for *TAS2R3* rs2270009 and *TAS2R4* rs2234001 as reported in GTEx.
ncRNA, non-coding RNA. Gene type information was obtained from GeneCards.

SNP	Reference allele	Alteration allele	Frequency of the alteration allele	Regulated gene	Gene type	*P*-value	Normalized effect size
rs2270009	C	T	0.46	*WEE2-AS1*	ncRNA gene	6.70E-67	0.76
				TAS2R4	Protein coding	6.30E-38	0.64
				TAS2R5	Protein coding	3.50E-10	-0.25
				PRSS37	Protein coding	6.30E-09	-0.34
				OR9A3P	Pseudogene	4.30E-08	-0.33
				TAS2R6	Pseudogene	4.40E-07	-0.28
				WEE2	Protein coding	0.0000026	0.31
rs2234001	G	C	0.49	*WEE2-AS1*	ncRNA gene	3.10E-62	0.74
				TAS2R4	Protein coding	3.70E-40	0.65
				TAS2R5	Protein coding	5.70E-13	-0.29
				TAS2R6	Pseudogene	2.00E-09	-0.33
				PRSS37	Protein coding	4.50E-09	-0.34
				OR9A3P	Pseudogene	5.10E-09	-0.35
				WEE2	Protein coding	0.000017	0.28

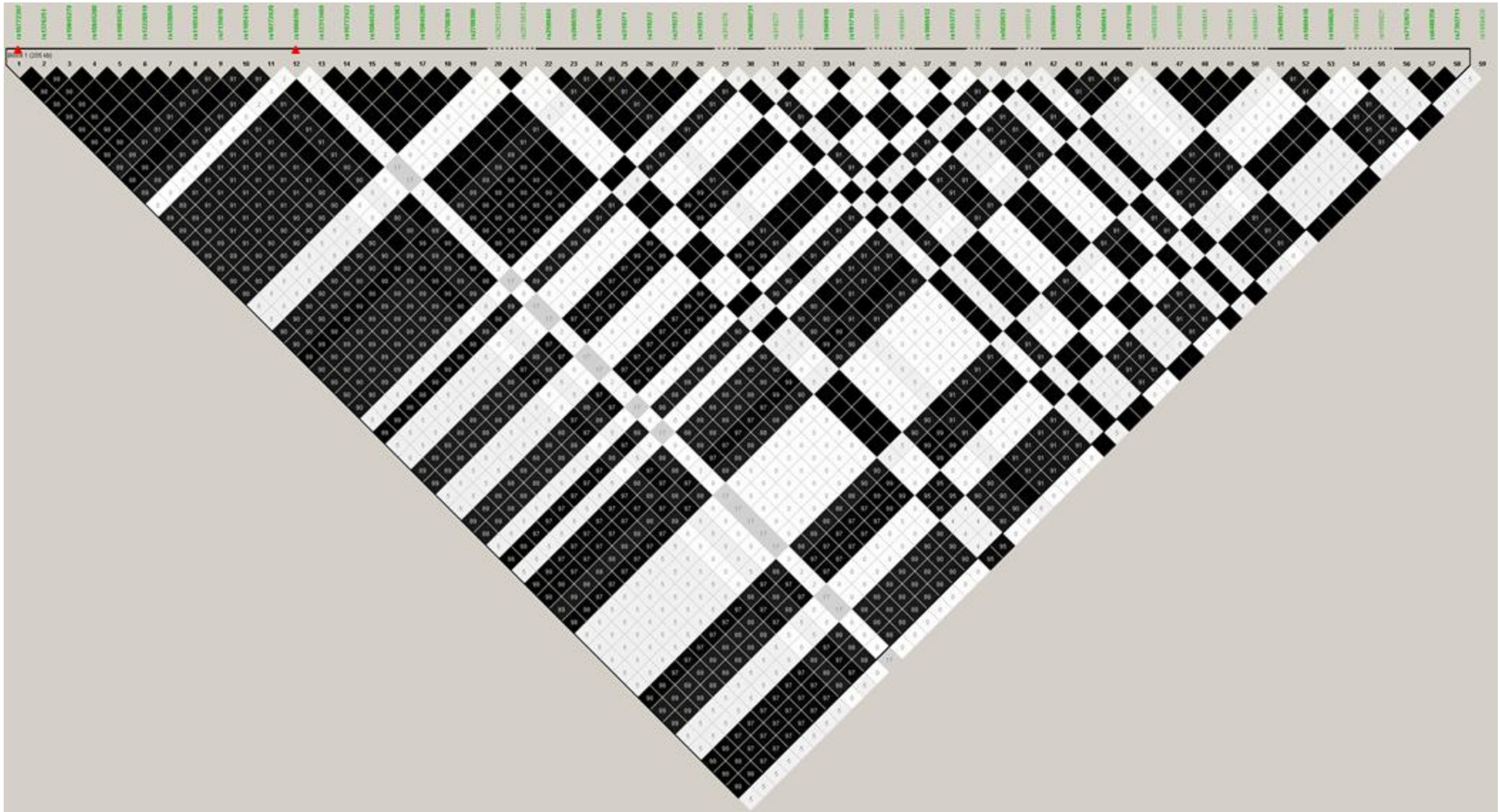

Supplementary Figure S1. Linkage disequilibrium patterns of *TAS2Rs* on chromosome 12.
Red triangles denote the tagging loci.
